# Silicon-Based Scaffold for Wound Healing Skin Regeneration Applications: A Concise Review

**DOI:** 10.3390/polym14194219

**Published:** 2022-10-08

**Authors:** Izzat Zulkiflee, Syafira Masri, Mazlan Zawani, Atiqah Salleh, Ibrahim Nor Amirrah, Mohd Farhanulhakim Mohd Razip Wee, Salma Mohamad Yusop, Mh Busra Fauzi

**Affiliations:** 1 Centre for Tissue Engineering and Regenerative Medicine, Faculty of Medicine, Universiti Kebangsaan Malaysia, Jalan Yaakob Latiff, Bandar Tun Razak, Cheras, Kuala Lumpur 56000, Malaysia; 2 Institute of Microengineering and Nanoelectrics, Universiti Kebangsaan Malaysia, Bangi 43600, Malaysia; 3 Department of Food Sciences, Faculty of Science and Technology, Universiti Kebangsaan Malaysia, Bangi 43600, Malaysia

**Keywords:** silicone, wound healing, tissue engineering, skin regeneration

## Abstract

Silicon has made its breakthrough in various industries, including clinical and biomedical applications. Silicon-based biomaterials that were fabricated into various types of scaffolds may attract interest due to their highly favorable properties covering their excellent biocompatibility, high surface area, mechanical strength, and selectivity depending on their application including film, hydrogel, nanoparticles, and so on. Silicon-based materials have also shown exciting results involving cell culture, cell growth, as well as tissue engineering. In this article, a simple review compromising the evaluation of silicon’s unique properties has been discussed and followed by the application of the silicone-based product in future perspectives in biomedical fields. The review goals are to widen and inspire broader interest in silicone-based materials in wound healing research.

## 1. Introduction

Tissue engineering (TE) is a scientific field that focuses on synthesizing tissue and organ replacements in the laboratory by manipulating cellular and biomechanical parameters. The tissue engineering triads consist of the cells that make up the tissue, the scaffold, which serves as a platform for the cells to proliferate and generate the physical form of tissue, and biological signalling pathways [1]. In contrast to the traditional medical therapy method, no cells are engaged in applying treatments. In general, the tissue engineering and regenerative medicine field are plagued by issues related to applications, production, efficacy, and functionality. The methods in this field may always be wondered about and questioned by other researchers to the fullest. The problems encountered are mainly regarding the sources and choices for the components of the materials used as well as limitations of one’s material.

Scaffolds are materials engineered to contribute to the formation of new tissues by giving rise to desirable cellular interactions for medical applications and purposes. The scaffold’s primary purpose is to resemble the extracellular matrix (ECM), which should support normal cell functions, especially the differentiation and proliferation of cells. The scaffold needs to be biocompatible, have the proper porosity and porous microstructure, and have the appropriate surface chemistry to support cell attachment, proliferation, and differentiation in order to serve its role in tissue engineering. The use of tissue engineering in reconstructive surgery has been broadened. Due to disconnects between experimental research and clinical practice, it currently faces some limitations for widespread clinical implementation.

From biomaterials and bioengineering perspectives, specifically in the early 2000s, it was improbable that synthetic polymers would be the best substrates for tissue-engineered skin [2]. Metcalfe and Ferguson [3] reviewed the relevant issues in a 2007 study whereby they found that no bioengineered skin at the time perfectly replicated the anatomy, biological stability, physiology, or aesthetic qualities of healthy skin, with significant issues under-vascularization, severe scarring, poor biocompatibility of the supporting membranes and a lack of complexity of differentiated structures. These issues were highlighted by Van Der Veen et al. [4], who emphasised that fibroblasts must be provided with binding sites to prevent the foreign body’s reaction to the degrading synthetic material from disrupting the wound healing process chemotactic signals that can guide cell behaviour. It is obvious that the collagen matrix is much more effective in eliciting these reactions than synthetic polymers. In a recent study on keratinocyte delivery in burn wound care, Ter Horst et al. [5] emphasised this point by referring the natural biopolymer hydrogels—like those made of chitosan, alginate, fibrin, and collagen—can act as supportive matrices in cell delivery, but not those made of synthetic polymers.

Natural scaffolds currently available ECM materials are still broadly used, including protein- and polysaccharide-based materials. Slowing down the rate of deterioration of these and other materials using cross-linking agents (such as glutaraldehyde and water-soluble carbodiimide) can be utilised. Although natural materials have good biocompatibility, there might occasionally be problems with possible immunogenicity [6]. More than 1 cm in diameter skin wounds that penetrate the dermis require specialized care since they do not heal or recover on their own, and they may leave behind substantial scarring that restricts joint motion and causes severe cosmetic deformities [7]. Autologous skin grafts continue to be the gold standard for major cutaneous wounds, a method that dates back several thousand years. [8]. The lack of sufficient residual unharmed donor sites from which to harvest skin graft material is the constraint for autologous skin transplantation. Although meshing the skin—a procedure in which the skin graft is uniformly perforated and stretched to cover larger areas of the wound—allows for the extension of coverage, the lack of dermis in the stretched meshed skin graft’s interstices and the slow epithelialization from graft margins across interstices lead to more significant graft contraction and a pronounced crocodile skin appearance of the scar.

In general, skin grafts may not work well in regions where wounds penetrate deeply into the dermis, and/or extensive scarring may result from the absence of a functional dermis. To overcome some of these restrictions, skin substitutes were first created. For instance, biodegradable matrix materials can simulate the dermis, and living cultured skin substitutes made from keratinocytes and fibroblasts procedures are now possible. Tissue-engineered skin products can serve as/for the following purposes: (a) a defence mechanism by constructing a mechanical barrier to microorganisms and vapour loss; (b) a delay by delivering some wound cover after early wound debridement until repeated skin transplants or cultured autologous cell applications may achieve permanent wound closure, especially in severe burns; (c) enhancing natural host wound healing responses by delivering to the wound bed dermal matrix components, cytokines, and growth factors; and (d) adding new structures to the wound that remain throughout and/or after wound healing, such as cultured cells or dermal collagen [9]. Even while none of the currently available treatments can entirely restore the missing skin, they have all been utilised to treat acute severe wounds, such as burns, as well as chronic non-healing wounds like diabetic ulcers and venous ulcers.

**Figure 1:**
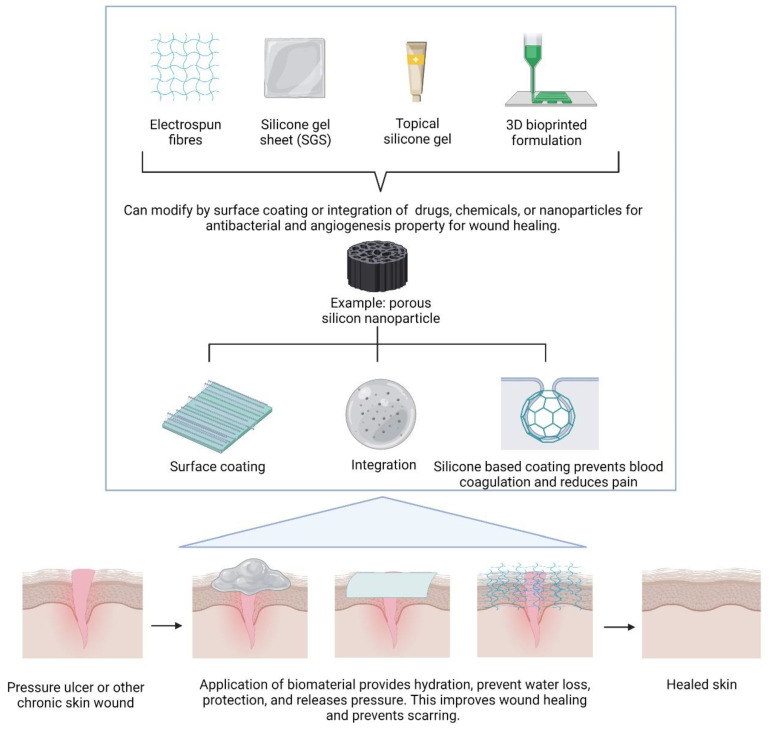
Some modifications and use of silicone-based biomaterials to different forms for the application of wound healing, created by Biorender.

The advancements in modern medical technology have led to the creation of numerous imaginative wound dressings. Different forms of dressings, such as films, foams, or hydrophilic gels, are made from various materials, such as natural source materials like chitosan, alginate, collagen, and gelatin, or synthetic materials such as PVA, silicon, or even silicone. These state-of-the-art wound dressings have some remarkable characteristics that improve wound healing. However, there are still a number of issues with these materials, including poor mechanical and physical properties that could cause infection, dehydration, or the maceration of wounds. Despite all the information above, an in-depth review of silicon and silicone as biomaterial use in regenerative medicine will be discussed further. The primary goal of this review is to examine how silicon, one of the key components of biomaterials for tissue engineering, may aid in the regeneration of skin and the healing of wounds. Figure 1 shows the overall idea of how silicone can be modified into different types of scaffolds and the application of the scaffold towards wound healing treatment.

## 2. Silicon as a Biomaterial

**Figure 2:**
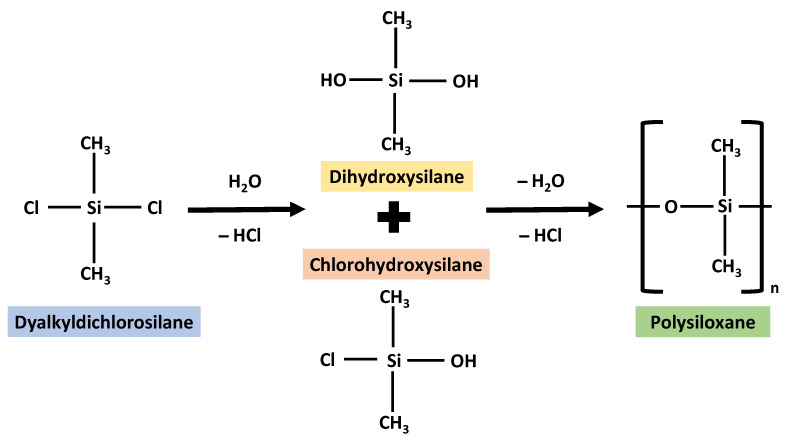
The synthesis of silicone redrawn and adopted with some modifications in reference from the image from Odian, G. (2004) [13].

Why is silicone chosen as the biomaterial for medical devices and regenerative medicine? Silicone polymers are versatile, and depending on the application, and they can be formed into various materials, such as elastomers, gels, adhesives, and more [10]. Silicone’s low glass-transition temperature of around −125 °C is one of its most notable characteristics, allowing the material to keep its flexibility even when exposed to severe temperatures, such as in cold storage. In terms of their advantage, silicones have the capacity to preserve mechanical qualities from −40 °C to 185 °C [10]. Silicone elastomers come in various hardness, are resistant to UV rays, have high heat, and are resistant to chemicals. They also have good electrical qualities, flame resistance and can be sterilised by steaming, autoclaving or gamma radiation [10]. Silicone is a gas-permeable, optically transparent, and a material that is simple to produce. Another factor that contributes to silicone’s appropriateness for long-term use is its hydrophobicity [11], hence providing optimal moisture content as the transdermal water loss (TWL) decreases when treated with silicone [12], which prevents encrustation. Furthermore, silicone adhesive has high gas and moisture permeability, adheres well to the skin, and does not irritate it. Depending on the use, silicone can be created in several different ways. In addition, silicone is different from silicon, which also plays an essential role in tissue engineering and regenerative medicine. Silicon is pure and generally seen as silica or silicon dioxide. In medicine, it is commonly used as an implant, bandage, dressing, and contact lens. Some of the applications refer to its different forms such as nanoparticles, porous sponges, hydrogel, films and fibres. Polysiloxane, famously know as silicone, can be synthesized by the hydrolysis of chlorosilanes with water, mixed with silanols (such as chlorohydroxysilane and dihydroxysilane) which will then undergo dehydration and dehydrochlorination [13]. The synthesis process can be referred in Figure 2 below. 

## 3. Silicon Forms and Applications

### 3.1. Nanoparticles

**Figure 3:**
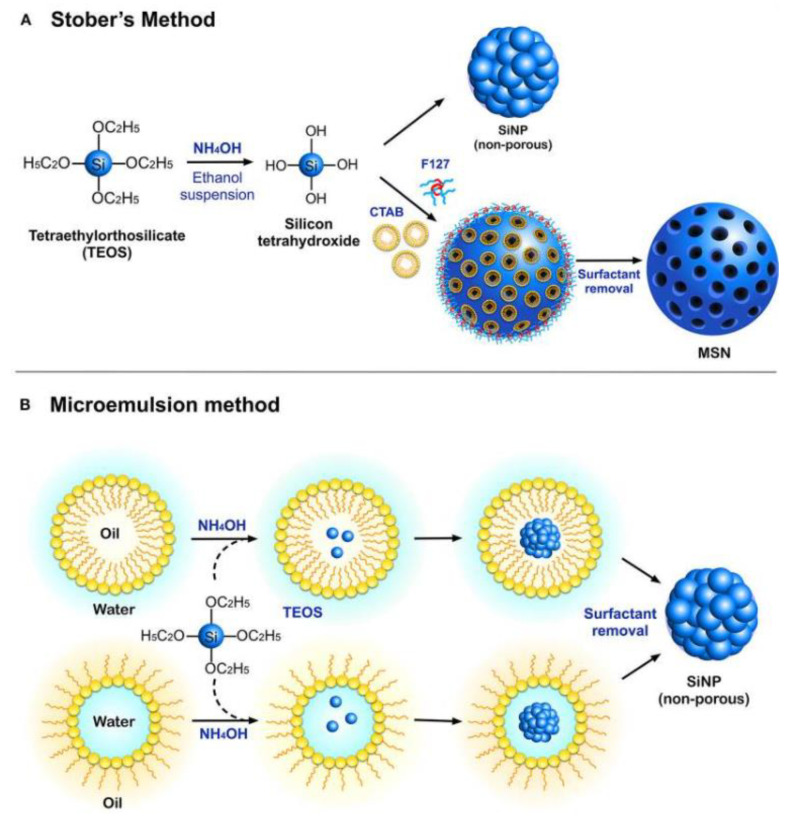
The synthesis of silicon nanoparticles using two different methods. [22]. Used under the Creative Common License.

The study of silicon nanoparticles is crucial to the field of nanotechnology. They have strong biocompatibility and are typically regarded as harmless. Silica nanowires can all disrupt bacterial cell reproduction, adhesion, and cell differentiation. These nanoparticles can help prevent the growth of biofilms. They have the potential to be utilised as carriers of antimicrobial agents in the application of biomedical fields. Ultimately, it may prevent bacterial infection during wound healing. Figure 3 shows the synthesis of silicon nanoparticles using two methods which are Stober’s method and the microemulsion method [13]. The production methods include physical, physico-chemical, chemical, electrochemical, and discretely sized Si nanoparticles [14]. It is significant to note that a recent study [15,16,17,18] has revealed that silicon-carbon nanohybrids (SiCNs), which are made of silicon nanoparticles (SiNPs) combined with functional carbon-related species like polymers, proteins, and DNA, are very promising for a variety of biological and biomedical applications because they have brighter fluorescence and better biocompatibility. Because of silicon’s exceptional biocompatibility, even small fluorescent SiNPs have been widely used as high-performance bioprobes for bioimaging and biosensing applications [19,20,21]. Silicon nanoparticles have shown tremendous potential for various biological and biomedical applications due to their various benefits, including but not limited to their exceptional mechanical, electrical, and optical capabilities and surface tailoring capacity. Since they can produce unique fluorescence at the nanoscale level, silicon nanoparticles (SiNPs), the most common silicon nanostructure, are very interesting.

**Figure 4:**
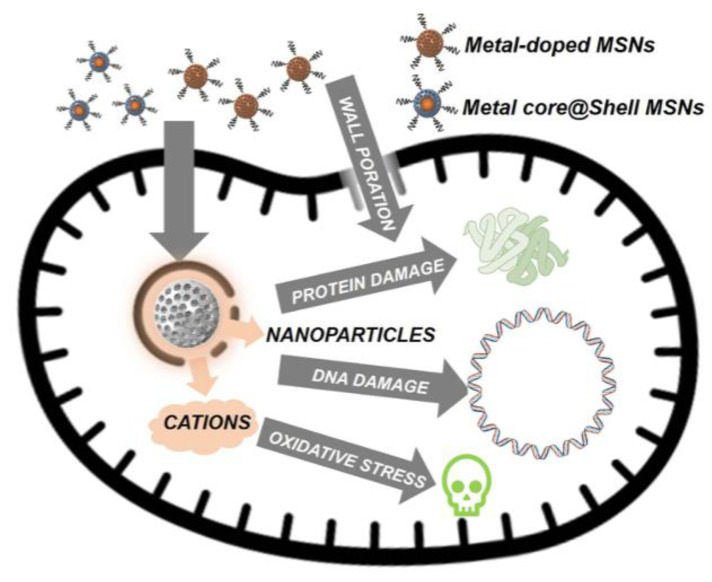
The mechanism of the action of the silicon nanocarrier delivering antibacterial material towards bacteria [23]. Creative Common Attribution—Non-Commercial (unported, v3.0). Licence (http://creativecommon.org/licenses/by-nc/3.0/, accessed on 1 August 2022).

Silicon nanoparticles can be a carrier for metallic nanoparticles such as silver to deliver to the targeted location. Silica nanoparticles (SiNPs) have emerged as a highly profitable option for a variety of medicinal uses, particularly in the treatment of cancer and microbial infections. SiNP has important benefits such a large surface area, simplicity in functionalization, and biocompatibility. Mesoporous silica nanoparticles (MSN), a porous version of the silicon pores, also offer additional benefits, including customizable pore size and volume, resulting in high drug loading capacity. Covalent and non-covalent ways of interaction with the payload have been made possible by tunable surface modifications of SiNPs, making it easier to control the release and activity of the functional payloads. Silicon nanoparticles have also shown good efficacy against biofilms, which justifies their use in implants and coatings for antibiofilm [22]. Figure 4 shows the mechanism of silicon nanocarrier delivering materials towards bacteria.

On the other note, as a representative of traditional Chinese medicine (TCM), a key player in wound healing, panax notoginseng has a broad range of pharmacological activities useful for anti-bacterial and wound closure. Additionally, its extractive has been used to treat internal and external bleeding brought on by wounds, and more recent studies have shown that notoginseng also has some promising anti-inflammatory, immunologic adjuvant, and anticancer properties. To relate to the above statements, a simple microwave-assisted synthesis method is used to manufacture a novel class of multifunctional SiCNs using herbal medicine, just like panax notoginseng [24]. Strong fluorescence of the SiCNs in their prepared form makes it possible to understand their pharmacological action and cell distribution. The SiCNs are particularly significant since they have specific antibacterial and wound healing activity, which means they could be employed to effectively treat wounds in vivo.

### 3.2. Fibres

Burns or diabetes-related wounds that are difficult to heal can benefit from the use of a wound dressing consisting of fibres of silica gel. During the healing process, the dressing completely absorbs into the body and serves as a support matrix for developing skin cells. An evaluation of orthosilicic acid-releasing fibre fleece (SIFIB) was done by Grotheer et al. [25], which shows that SIFIB is stable in shape, neutral in pH, and bioresorbable. SIFIB stays in the body after application and eventually breaks down without leaving any residue. Even while SIFIB followed the wound-healing process with granulocytic diapedeses into the dermal tissue, SIFIB had no negative effects on the wound’s ability to heal in an animal with a healthy body. Compared to sterile but otherwise treated control wounds, the wound area decreased, and the wound closed considerably faster on day 28. The silica fibre architectures may serve as conducting channels for cell migration, propagation, or spreading, respectively, within the granulation tissue of the healing wound, according to the results of the histological analysis of skin samples. Another advantage of rapid cutaneous regeneration may be this SIFIB characteristic. Other than that, results acquired in vitro study with primary human cell cultures that reveal that SIFIB did not exhibit any harmful effects with human skin-derived cell cultures allowing for the deduction that SIFIB has enormously good wound healing-relevant potential [26].

### 3.3. Hydrogels

It is now widely acknowledged that a wound bed needs to stay moist for better healing [27]. To minimise overhydration, a material must offer a moist environment while yet enabling a suitable moisture vapour transmission rate. Other than that, although inadequate epidermal migration is a defining characteristic of non-healing wounds, silicone’s ability to modulate electrical charges and its bacteriostatic properties may help improve wound healing by encouraging epidermal migration. A study was conducted to describe four cases in which the supplementary use of silicone gel allowed wounds to heal by secondary intention (SI) after skin tumour removal in order to epithelize more quickly [28]. Elderly patients with tumours on their scalps or extremities participated in the trial. All wounds were closed using an internal purse string suture, silicone gel, and a paraffin gauze dressing that was customary for the facility before being allowed to heal by SI. Therefore, the use of silicone gel in conjunction with a purse-string closure improved SI healing. This study may have limitations due to its retrospective character, but despite these, it offers some preliminary evidence in favour of the use of silicone gel as a low-cost supplement to SI healing. Topical silicone gel proved as successful in preventing scarring, albeit the benefits might not become apparent for about six months following surgery. In the treatment of scars, silicone gel’s effectiveness and safety profile were comparable to those of silicone gel sheets [29]. Other than that, 35 skin biopsies samples were examined under a microscope by Zappi et al. [30]. They found that 25 patients received liquid injectable silicone (LIS) for treating face scars between the ages of 1 and 23. The silicone droplets that showed silicone’s inertness and excellent permanence had no discernible adverse effects. An in vivo study by Yang et al. [31] reported that silicone gel application on rats’ skin reduces the inflammatory response and increases the healing index. In another study, Di Sun et al. successfully proved that their silicone gel possessed outstanding angiogenic, anti-inflammatory, and wound healing properties [32].

### 3.4. Films

An example of a commercial product of silicone in the form of film is Mepilex AG from Sweden. A synthetic wound dressing called Mepilex AG is frequently used to treat burn wounds with a partial thickness. It has three layers: a silicone layer covering the wound, an absorbent polyurethane foam layer, and a waterproof protective film that maintains the moistness of the wound environment while enabling gases to pass through the dressing. It also provides accelerated wound healing with the combination of antimicrobial properties, which is silver [33,34]. Besides that, a biosynthetic skin called Biobrane^TM^ is made of an inner nylon mesh that is adhered to a silicone membrane on the outside. It is possible for gases to pass through this dressing, but not liquids or bacteria. This keeps the burn area moist, which is vital in improving healing. However, great care must be taken not to cover infected wounds or those with eschar or debris. This technique is ideal for partial thickness wounds since the wound must also have sensibility and proper capillary blanching and refill. Additionally, Biobrane^TM^ (UDL Laboratories, Rockford, IL, USA) [35], a biosynthetic substance used in burn treatment, is made of silicone film attached to a nylon matrix that has been crosslinked with porcine collagen.

**Figure 5:**
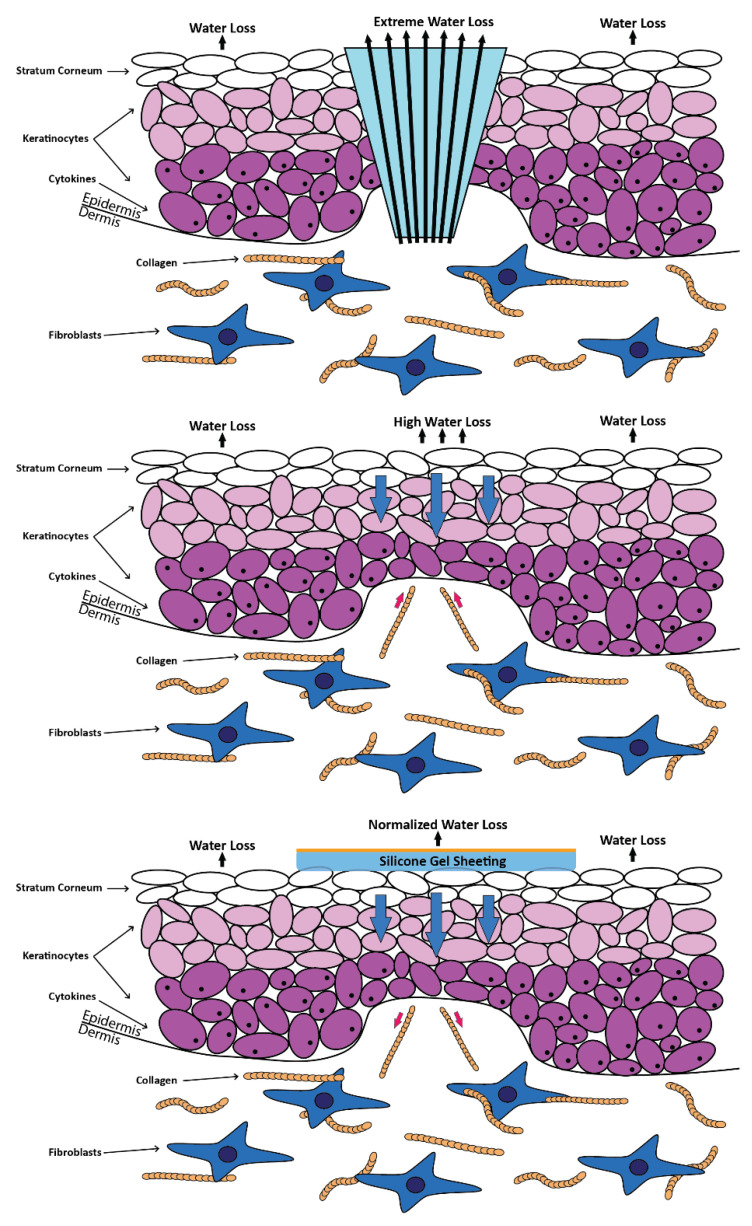
Application of silicone gel sheeting on top of the wound. The image was redrawn with a slight modification using Illustrator in reference to the figure from Bleasedale et al. [39].

Since Biobrane^TM^’s composition enables blood in the wound to clot naturally, attaching to the wound bed while re-epithelialization takes place, it is appealing to use it in burns. The outer silicone layer improves the wound environment by minimizing water loss through evaporation. Similar to Biobrane^TM^, Advanced Wound Bioengineered Alternate Tissue (AWBAT) [36] was approved by the Food and Drug Administration (FDA) in 2010. By making the silicone membrane more permeable and avoiding the use of harmful crosslinking agents to create a covalent bond between collagen peptide and the silicone-nylon composite, the limitations in Biobrane^TM^ have been improved in this product [37]. The first silicone therapy to treat keloids and hypertrophic scars was liquid silicone, developed in the 1970s. Topical silicone sheets were then introduced in the early 1980s. Its therapeutic effectiveness and efficacy seem to be secured and productive. Topical silicone sheet (TSS) application is currently frequently used to treat hypertrophic scars, with the ideal treatment time and technique by applying the topical silicone sheet for a minimum of three months, four hours per day [38]. Figure 5 shows an illustration of silicone gel sheeting [39].

### 3.5. Porous Sponges

The customizable porosity and surface chemistry of luminescent porous silicon (LuPSi) and its biocompatibility and degradability make it a potential material for biomedical applications [40]. These properties are proposed for the application of LuPSi as drug delivery and optical biosensors for bioimaging. Numerous studies have shown that the surface chemistry of LuPSi, particularly the oxidation of the porous silicon surface, strongly correlates with its luminous ability [41,42,43]. For instance, a sensing method to identify ROS in biological samples can be done by oxidation-triggered fluorescence recovery from dye-modified PSi. Numerous studies have shown that the oxidation of the PSi surface, particularly, is closely associated with the luminous feature of LuPSi [44]. Porous silicon (pSi) is a biomaterial appealing for application in vivo and ex vivo due to several distinctive characteristics. Bulk crystalline silicon is anodized in hydrofluoric acid (HF) solution to produce the biocompatible and high surface area pSi [45]. Numerous porosity layouts can be produced by altering the wafer resistivity, HF concentrations, and applied current densities. Pore diameters can be changed from nanometers to microns, producing surfaces with up to 800 m^2^g^−1^ of surface area. Depending on the pore size and chemistry, pSi can degrade over a period of days to months, producing the innocuous acid silicic acid. Additionally, pSi has shown in vivo biocompatibility when injected into the vitreous of rabbit eyes and the subconjunctival region of rats.

The biodistribution of pSi nanoparticles (pSi NPs) delivered orally, subcutaneously and intravenously has also been reported by Bimbo et al. [46]. They have also shown that the pSi NPs do not cause inflammatory responses or toxicity effects while demonstrating great in vivo stability in rats. It has also been shown that pSi can store and release different payloads of small-molecule medicines, oligonucleotides, or even protein therapies. Optical biosensors for detecting cancer, infectious diseases, and chronic wounds are among the ex vivo applications for the substance. These sensors take advantage of the material’s special optical and photonic capabilities. A study by Xu et al. [47] reported that silicone rubber membrane covered cutaneous wounds and aided in wound healing as it had the bionic structure of human skin, was non-toxic, and had excellent biocompatibility. The outcomes suggested that the prepared microporous silicone rubber membrane bilayer (SRM-B) might be used as a dressing for wounds as it shows potential as a fresh method of treating cutaneous wounds.

## 4. Silicon-Based Biomaterials in Wound Healing

**Figure 6:**
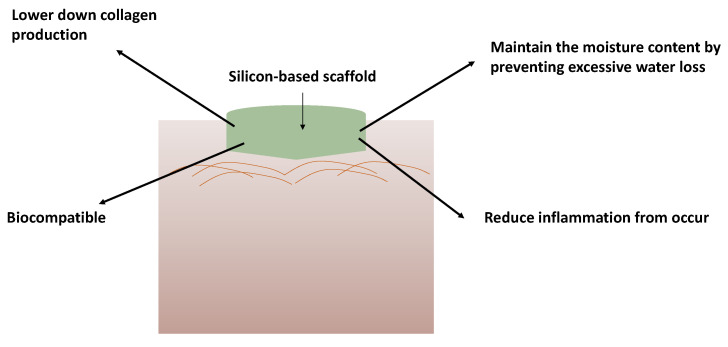
Application and the benefits of silicon-based scaffold on wounds for wound healing treatment.

The presence of polar functional groups on synthetic polymer-based scaffolds, such as carbonyl, carboxyl, ester, amino, and/or amide, raises the polar component of the surface’s free energy, making these biomedical devices more wettable and therefore more vulnerable to interactions with extracellular matrix (ECM) biomolecules [48]. Basically, it mimics the histological structure of the skin to help in preventing rejections such as allergies. It is important to note how the cell proliferates and is viable during the interaction of the synthetic material mentioned, silicon. Figure 6 generally implies how the application of silicon-based scaffold on top of wound and its beneficial effects. However, the exact mechanisms are still unknown. Therefore, cell signalling investigations are valuable for future perspective studies. A study by Tolksdorf et al. [49] discovered that different pore size and structure of silicone implants significantly alter the behaviours of the cell. Different conditions of the surrounding of the cells can vary in responses. Thus, a different trend of gene expression analysis was discovered; however, it still concludes that it is biocompatible towards human dermal fibroblasts. The cell proliferation and cell viability towards silicon-based biomaterials were discussed further below.

### 4.1. Cell Proliferation

Cell proliferation is important as it is one of the phases, especially the proliferation phase, in wound healing. This is the phase where the wound is rebuilt with new tissue made up of other extracellular matrixes such as collagen. It is also important to note that the proliferation phase is where the contraction of the wound occurs as the new tissues are built. Human skin fibroblasts’ proliferation and growth were not significantly affected by SIFIB, although it greatly slowed their growth in the late (>10 days) culture phase [50]. In the first seven days, the human acute monocytic leukaemia cell line (THP-1) SIFIB dramatically slowed down cell proliferation. After that, it was possible to see THP-1 cells growing normally and sometimes even significantly faster when the silica fleeces are present. In contrast, SIFIB-induced human skin keratinocytes showed a considerable and almost total suppression of growth. In a bone related study, a report by Han et al. [51] stated that Si ions increased BMSC proliferation, mineralisation nodule formation, and bone-related gene expression at a certain concentration.

Another study by Shie et al. [52] stated that an appropriate Si concentration was successful in boosting osteoblast-like cell proliferation and actively generating a biological response in MG63 cells via bone-specific protein synthesis. In relation to the skin, it has been hypothesised that silicon is crucial for optimum collagen synthesis as well as for activating the hydroxylation enzymes, which are crucial for the creation of the collagen network and improve skin elasticity and strength. Additionally, silicon is involved in the production of glycosaminoglycans [53,54]. Bhuyan et al. [55] reported that cells proliferate in an excellent manner and showed normal morphology, indicating a good biocompatibility.

### 4.2. Cell Viability

As for cell viability, silica-induced cell death was not the source of this negative effect on keratinocyte growth because SIFIB had no appreciable influence on the human skin keratinocytes viability. Like keratinocytes, endothelium and fibroblast cells were not significantly negatively affected by silica in terms of survival. On the other hand, when these cultures were stained with propidium iodide, it was evident that there were far more necrotic events than there were in the controls. ICP-MS measurements show that silica uptake significantly increased in THP-1 cultures as necrotic cell death progressed [25].

## 5. Limitations

One major drawback of using silicone is the cost of producing silicone materials. Silicone is a thermoset material that requires curing in order to meet biocompatibility and application-specific requirements. It cannot be changed once it has been treated. The curing process necessitates a precise temperature, pressure, and time balance, as well as additional equipment and curing catalysts. As a result, prominent silicone OEMs in the United States typically advertise lead times of at least 8 to 12 weeks. The curing process can add up to a lot of money, and there are usually opportunity costs associated with the long development time. Other than that, silicones exposed to extreme temperatures can be quite strong and stable; however, its endurance is substantially reduced by wear and tear—and silicone is particularly prone to this type of external damage. Repetitive action and heavy rolling equipment in a fast-paced hospital or clinic provide enough opportunities for silicone cables to be damaged in this way.

An allergic response is another major reason someone might wish to stay away from silicone-containing products. Although the research found no substantial negative effects, a person may still be allergic to silicone or any other substance, just like they may be to any other product. Itching, burning, redness, hives, and swelling are symptoms of an allergic reaction. A preliminary test such as performing a patch test and dabbing a small bit of a new product (silicone) on their arm before using it on their skin to ensure that no adverse reaction forms. In addition, silicone gel therapy showed a good result in improving wound healing and scar treatments. However, it needs to be reapplied and wear constantly for 6 to 12 months to achieve an optimum result.

## 6. Conclusions

Depending on their purpose, silicone polymers can be made into a wide range of diverse material types, including adhesives, fillers, elastomers, gels, and many more. It is worth mentioning silicone biomaterial interactions with host tissue and their inert, chemically stable, and long-lasting properties. Silicone-based biomaterials have a promising future if device manufacturers continue to screen and acquire their raw ingredients responsibly. Because silicon functions in a variety of human health issues and possesses aesthetic qualities, research on the usage of silicon-containing supplements reveals that this element has significant therapeutic potential. Furthermore, silicone has a significant role in the medical industry’s future, thanks to the development of silicone-based 3D bioprinting and tissue engineering technologies. Furthermore, ensuring that the implants are safe and biocompatible and that the material is of good quality and well-regulated to avoid adverse reactions remains a difficulty.

## Data Availability

The data presented in this study are available on request from the corresponding author.
